# The gene *transformer-2 *of *Anastrepha *fruit flies (Diptera, Tephritidae) and its evolution in insects

**DOI:** 10.1186/1471-2148-10-140

**Published:** 2010-05-13

**Authors:** Francesca Sarno, María F Ruiz, José M Eirín-López, André LP Perondini, Denise Selivon, Lucas Sánchez

**Affiliations:** 1 Centro de Investigaciones Biológicas (C.S.I.C.), Ramiro de Maeztu 9, 28040 Madrid, Spain; 2 CHROMEVOL-XENOMAR Group, Departamento de Biología Celular y Molecular, Universidade da Coruña, 15071 A Coruña, Spain; 3 Departamento de Genética e Biologia Evolutiva, Instituto de Biociências, Universidade de São Paulo, 05508-0900 Sao Paulo, Brazil

## Abstract

**Background:**

In the tephritids *Ceratitis*, *Bactrocera *and *Anastrepha*, the gene *transformer *provides the memory device for sex determination via its auto-regulation; only in females is functional Tra protein produced. To date, the isolation and characterisation of the gene *transformer-2 *in the tephritids has only been undertaken in *Ceratitis*, and it has been shown that its function is required for the female-specific splicing of *doublesex *and *transformer *pre-mRNA. It therefore participates in *transformer *auto-regulatory function. In this work, the characterisation of this gene in eleven tephritid species belonging to the less extensively analysed genus *Anastrepha *was undertaken in order to throw light on the evolution of *transformer-2*.

**Results:**

The gene *transformer-2 *produces a protein of 249 amino acids in both sexes, which shows the features of the SR protein family. No significant partially spliced mRNA isoform specific to the male germ line was detected, unlike in *Drosophila*. It is transcribed in both sexes during development and in adult life, in both the soma and germ line. The injection of *Anastrepha transformer-2 *dsRNA into *Anastrepha *embryos caused a change in the splicing pattern of the endogenous *transformer *and *doublesex *pre-mRNA of XX females from the female to the male mode. Consequently, these XX females were transformed into pseudomales. The comparison of the eleven *Anastrepha *Transformer-2 proteins among themselves, and with the Transformer-2 proteins of other insects, suggests the existence of negative selection acting at the protein level to maintain Transformer-2 structural features.

**Conclusions:**

These results indicate that *transformer-2 *is required for sex determination in *Anastrepha *through its participation in the female-specific splicing of *transformer *and *doublesex *pre-mRNAs. It is therefore needed for the auto-regulation of the gene *transformer*. Thus, the *transformer/transfomer-2 > doublesex *elements at the bottom of the cascade, and their relationships, probably represent the ancestral state (which still exists in the Tephritidae, Calliphoridae and Muscidae lineages) of the extant cascade found in the Drosophilidae lineage (in which *tra *is just another component of the sex determination gene cascade regulated by *Sex-lethal*). In the phylogenetic lineage that gave rise to the drosophilids, evolution co-opted for *Sex-lethal*, modified it, and converted it into the key gene controlling sex determination.

## Background

Sex determination refers to the developmental programme that commits the embryo to following either the male or the female pathway. The past few years have seen a great amount of interest in the evolution of developmental mechanisms at the genetic and molecular levels, and in determining the evolutionary processes by which these mechanisms came into existence. In this respect, sex determination is a process that seems to be exceptionally suitable for comparative study, given the great variety of mechanisms that exist. Indeed, sex determination has long been of major interest not only as a developmental process but also because of the evolutionary problem it poses - a problem that can only be solved by identifying and comparing the genetic structures of sex determination pathways. Molecular genetic technologies now allow such comparisons to be made. In addition, sex determination in the reference system of insects - that of *Drosophila melanogaster *- is known in fine detail, making truly informative comparisons possible.

The characterisation of the sex determination genes in *D. melanogaster *has shown that their control during development is governed by the sex-specific splicing of their products (reviewed in [1]). The product of a gene controls the sex-specific splicing of the pre-mRNA from the downstream gene in the genetic cascade. *Sex-lethal (Sxl) *is at the top of this cascade and acts as the memory device for female sexual development via its auto-regulatory function: its product controls the splicing of its own pre-mRNA [2,3]. In addition, Sxl controls the splicing of the pre-mRNA from the downstream gene *transformer (tra) *[4-6]. The Tra product and the product of the constitutive gene *transformer-2 (tra-2) *control the sex-specific splicing of pre-mRNA from the gene *doublesex (dsx) *[7-10], which is transcribed in both sexes but gives rise to two different proteins, DsxF and DsxM [11,12]. These are transcription factors that impose female and male sexual development respectively via the sex-specific regulation of the so-called sexual cytodifferentiation genes.

Genes homologous to the sex determination genes of *D. melanogaster *have been sought in other insects (reviewed in [13]). In the tephritid fruit flies, the gene *Sxl *has been characterised in *Ceratitis capitata *[14] and in *Bactrocera oleae *[15], *tra *has been characterised in *C. capitata *[16], *B. oleae *[17] and in twelve *Anastrepha *species [18], and *dsx *has been characterised in *C. capitata *[19], *B. oleae *[15] and in eleven *Anastrepha *species [20,21].

The tephritid *Sxl *gene is not regulated in a sex-specific fashion, and therefore the same *Sxl *transcript encoding the functional Sxl protein is found in both males and females [14,15]. Thus, in the tephritids, *Sxl *does not appear to play the key discriminating role (memory device) in sex determination that it plays in *Drosophila*.

As in the drosophilids, the tephritid *tra *gene is constitutively expressed in both sexes and its primary transcript shows sex-specific alternative splicing. However, whereas in the drosophilids *Sxl *regulates *tra*, in the tephritids this gene appears to have an auto-regulatory function that produces functional Tra protein specifically in females. The gene *tra *in the tephritids has male-specific exons that contain translation stop codons. The incorporation of these exons into the mature *tra *mRNA in males determines that, in this sex, a truncated, non-functional Tra protein is produced. In females, the male-specific exons are spliced out because of the presence of Tra protein [16-18]. The presence of putative Tra-Tra2 binding sites in the male-specific exons and in the surrounding introns may suggest that the Tra2 protein participates in the *tra *auto-regulatory function. The introduction of *Ceratitis *[16] or *Bactrocera *[17]*tra*-dsRNA into *Ceratitis *or *Bactrocera *embryos, respectively, results in the destruction of endogenous *tra *function in both species and the subsequent complete transformation of females into pseudomales. Together, these results support the proposal of Pane *et al*. [16] that the key regulatory role played by *tra *is to act as the memory device for sex determination via its auto-regulatory function.

The tephritid gene *dsx *codes for male- and female-specific RNAs, which encode the male-specific and female-specific Dsx proteins [15,19-21]. Putative Tra-Tra2 binding sites have been found in the female-specific exon, suggesting that, as in *Drosophila*, male-specific splicing represents the default mode and that female-specific splicing requires Tra protein, which would only be present in females.

So far, the isolation and characterisation of the gene *transformer-2 (tra-2) *in the tephritids has been only performed in *C. capitata *[22,23]. This gene is transcribed in both sexes. The injection of *Ceratitis tra-2 *dsRNA into *Ceratitis *embryos results in the transformation of genotypically female embryos into adult pseudomales, highlighting the role of *tra-2 *in *Ceratitis *sex determination [23]. Its function is required for the female-specific splicing of *dsx *and *tra *pre-mRNA; it therefore participates in *tra *auto-regulatory function [23].

The study of the evolution of the sex determination gene cascade (i.e., the genes and their interactions) requires its characterisation in different species. To better analyse the evolution of the gene *tra-2*, and more specifically its pivotal role in *tra *auto-regulation in the tephritids, its characterisation was undertaken in eleven tephritid species belonging to the less extensively analysed genus *Anastrepha*. The present analysis therefore included *Anastrepha obliqua, A. amita *and *A. sororcula*, plus the four closely related species of the so-called *Anastrepha fraterculus *complex - *A*. sp.1 *aff. fraterculus, A*. sp.2 *aff. fraterculus, A*. sp.3 *aff. fraterculus *and *A*. sp.4 *aff. fraterculus *[24,25], all of which belong to the *fraterculus *group [26] - along with *A. serpentina *(*serpentina *group), *A. striata *and *A. bistrigata *(*striata *group) and *A. grandis *(*grandis *group) [26].

Firstly, the gene *tra-2 *in the reference species *A. obliqua *was isolated and its molecular organisation, expression pattern and encoded product studied. Secondly, the *tra-2 *ORFs in the other *Anastrepha *species were identified, and a comparative analysis of all the known insect Tra2 proteins undertaken. Thirdly, its function in sex determination was studied by following the sexual development (at the morphological, chromosome and molecular levels) of *Anastrepha *embryos in which endogenous *tra-2 *function was destroyed by the injection of *tra-2 *dsRNA. Finally, the phylogeny of *tra-2 *in these species and in other insects was investigated.

## Results

### The molecular organisation of *tra-2 *in *Anastrepha obliqua*, and its expression

**Figure 1:**
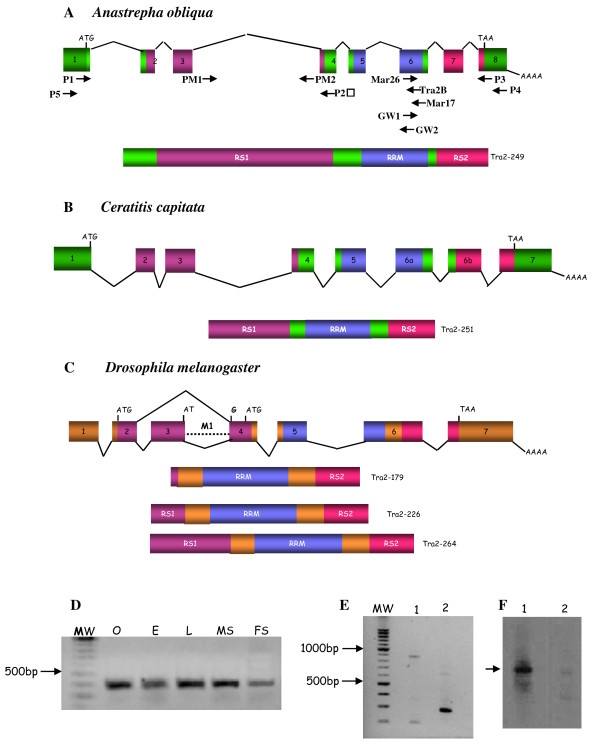
**Comparison of the molecular organisation of the *tra-2 *of *A. obliqua *(A), *C. capitata *(B) and *D. melanogaster *(C) and their proteins**. Exons (boxes) and introns (dashed lines) are not drawn to scale. The numbers inside the boxes indicate the number of the exon. The beginning and the end of the ORF are indicated by ATG and TAA respectively. AAA stands for poly-A(+). **(D) **Expression of gene *tra-2 *of *A. obliqua*. RT-PCR analyses of total RNA from ovaries (O), embryos (E), from male and female larvae (L), male soma (head plus thorax, MS) and female soma (head plus thorax, FS). **(E) **Expression of gene *tra-2 *of *A. obliqua*. RT-PCR analysis of total RNA from *A. obliqua *testis. Lane 1 corresponds to PCR with primers PM1 and P2; lane 2 corresponds to PCR with primers P1 and P2 (see location of primers in Figure 1A). **(F) **Southern-blot corresponding to the gel shown in (E) and hybridisation with a probe specific for intron 3 of *A. obliqua*. The arrow marks the hybridisation to the higher band in lane 1 of Figure 1E. The size of the mRNAs encoding the proteins shown in this figure are: 1923 bp for *A. obliqua *mRNA, 1113 bp for *C. capitata *mRNA, and 960, 1583 and 1391 bp for *D. melanogaster *mRNAs *tra2-179, tra2-226 and tra2-264*, respectively.

The first step in the isolation of the *A. obliqua tra-2 *gene (*Aotra2*) was to perform RT-PCR on total RNA from female adults. Reverse transcription was performed using the primer oligo-dT, while two nested PCR reactions were performed with three degenerated primers: the nested forward Mar17 and Mar26 primers, and the reverse Tra2-B primer, the latter located in the very well conserved RRM domain of the Tra2 protein (see Materials and Methods and Figure 1A). The first PCR reaction was performed using the pair of primers Mar17 plus Tra2B, the second using Mar26 plus Tra2B. An amplicon of 92 bp was amplified, cloned and sequenced. The conceptual amino acid sequence of this amplicon showed a high degree of similarity with the 3' region of the RRM domain of *D. melanogaster *Tra2 protein, indicating that a fragment of the putative AoTra2 protein had been isolated.

To determine the molecular organisation of *Aotra2 *the following strategy was followed. Firstly, 3'- and 5'-RACE analyses were performed. To this end, specific primers from the amplified sequenced were synthesised (see Materials and Methods). These primers were used in nested PCR reactions involving total RNA from male and female adults. The amplicons, which were the same in both sexes, were then cloned and sequenced.

A GenomeWalker library of *A. obliqua *was synthesised (see Materials and Methods) and used to perform PCR genome-walking on the genomic DNA of *A. obliqua *from the initial amplicon towards the 5' and 3' directions (see Materials and Methods for the primers used). The genomic amplicons were cloned and sequenced. The sequences of the genomic fragments thus generated were compared with the *A. obliqua *male and female cDNA sequences previously determined. In this way, the exon/intron junctions were unambiguously identified.

Figure 1A shows the molecular organisation of *Aotra2 *and its comparison with that of *C. capitata tra-2 *(Figure 1B) and *D. melanogaster tra-2 *(Figure 1C). The transcription unit of *Aotra2 *was made up of 3635 bp and composed of eight exons and seven introns. The putative translation start site was the last three nucleotides of exon1, and the stop codon was located at 15 nucleotides of the beginning of exon 8. The size of the exons and introns of *tra-2 *from *Anastrepha, Ceratitis *and *Drosophila *is given as additional File 1. The sizes of the mRNAs encoding the Tra2 proteins shown in Figure 1 are given in the legend to this Figure.

The molecular organisation of gene *tra-2 *in *Anastrepha, Ceratitis *and *Drosophila *was very similar. The conceptual translation of the *Aotra2 *mRNA encoded a protein with an RNA-recognition motif (RRM) flanked by two arginine-serine rich regions (RS) as in *Ceratitis *and *Drosophila*. The RS1 domain was encoded in exons 2, 3 and 4, the RRM domain in exons 5 and 6, and the RS2 domain in exons 7 and 8 in *Anastrepha *and 6b and 7 in *Ceratitis *and *Drosophila*. The putative Aotra2 protein should have some additional amino acids at the amino-terminal end preceding the RS1 domain.

In *D. melanogaster*, *tra-2 *gives rise to three mRNAs (*tra2-226, tra2-264 *and *tra2-179*) by alternative splicing pathways and alternative promoters, which encode three distinct isoforms of the Tra2 protein [27,28]. As in the case of other dipterans *C. capitata *[23], *Musca domestica *[29] and *Lucilia cuprina *[30], only a single *tra2 *mRNA was detected in *A. obliqua*. This was confirmed by overlapping PCR on total RNA of males and females using the primers shown in Figure 1A.

The expression of *Aotra2 *was studied by performing RT-PCR on total RNA from a mixture of male plus female embryos, from a mixture of male plus female larvae at different developmental stages, from the heads plus thoraces of male and female adults (separately), and from adult ovaries. The primers used were GW1 from exon 1 and P4 from exon 8 (see Figure 1A). In all cases, a fragment of 368 bp was amplified (Figure 1D). This was cloned and sequenced confirming that it corresponded to the expected *Aotra2 *fragment. Negative controls in all these PCR reactions produced no amplicons (see Materials and Methods). These results indicate that the gene *Aotra2 *is expressed at all developmental stages and during adult life in both sexes, including the ovaries of adult females, what suggests that *tra-2 *has a maternal expression.

In the male germ line of *D. melanogaster*, the Tra2 protein acts negatively on the splicing of its primary transcript promoting the inclusion of intron M1 in the mRNA (see Figure 1C), which encodes a truncated, non-functional Tra2 protein [31]. This aberrant mRNA comprises about 50% of the total *tra2 *mRNA in the male germ line [32]. This retention of the M1 intron is the mechanism by which the functional Tra2 protein limits its own synthesis since the final amount of this protein is crucial for male fertility [33]. This negative regulation of the Tra2 protein is exerted by its binding to specific ISS-sequences located in intron M1 [34]. The existence of putative Tra2-binding ISS sequences in intron 3 of *Aotra2 *gene (data not shown) prompted us to investigate whether the mRNA isoform carrying this intron is also significantly produced in the testis of *A. obliqua*, as a sign that *tra-2 *negative auto-regulation might exist in the *A. obliqua *male germ line. To this end, RT-PCR was performed on total RNA from adult testis using the pair of primers P1 from exon 1 and P2 from exon 4. Only a single fragment of 300 bp was amplified that corresponded to the mature mRNA lacking introns 1, 2 and 3 (Figure 1E, lane 2). Notwithstanding, when primers PM1 at the beginning of intron 3 and primer P2 from exon 4 were used for PCR, two bands of about 200 and 900 bp, respectively, were amplified (Figure 1E, lane 1). Negative controls for all these PCR reactions produced no amplicons (see Materials and Methods). The 900 bp fragment might correspond to an mRNA isoform retaining intron 3. To confirm this expectation, a Southern-blot with the RT-PCR products of the two PCR reactions shown in Figure 1E was carried out using a probe corresponding to intron 3 (see Materials and Methods). Only the 900 bp band - not the small 200 bp band - in lane 1 showed positive hybridisation. The 300 bp band in lane 2 did not hybridise as expected since this corresponds to an mRNA lacking intron 3 (Figure 1F). Collectively, these results indicate that the mRNA isoform retaining intron 3 in the male germ line of *A. obliqua *is very poorly represented, in contrast to that seen in *Drosophila *and in agreement with which occurs in *C. capitata *[23] and *Musca domestica *[29].

### The Tra2 protein of *A. obliqua *and other *Anastrepha *species

The conceptual translation of the female *Aotra2 *mRNA showed it to encode a polypeptide of 249 amino acids. It shared the main structural features that characterise the SR protein family, i.e., the RNA-binding motif (RRM) and two RS-domains, which are rich in serine-arginine dipeptides and confer upon these proteins the capacity to interact with others.

To characterise the Tra2 protein of other *Anastrepha *species, it was assumed that the *tra-2 *gene of the *Anastrepha *species studied here had a molecular organisation similar to that of *A. obliqua*. Under this assumption, RT-PCR analyses of total RNA from female adults were performed. Reverse transcription was performed with the P4 primer located in the 3'UTR region of *Aotra2*. PCR amplification of the cDNA was undertaken using the pair of primers P5 (from the 5'UTR of *Aotra2*) plus P4 (see Figure 1A). In this way the whole ORF of the *tra-2 *gene of all *Anastrepha *species studied here was amplified, cloned and sequenced. The Tra2 protein of all these *Anastrepha *species was composed of 249 amino acids. Their degree of similarity (i.e., identical plus conserved amino acids) was extremely high, ranging from 97.2 to 100% (see additional File 2).

The putative Tra2 protein from *A. obliqua *(used as the reference species), those from the dipterans *C. capitata*, *B. oleae*, *M. domestica, L. cuprina, D. melanogaster, D. virilis *and *D. pesudoobscura*, that from the lepidopteran *Bombyx mori *(the silkworm) and from the hymenopteran *Apis mellifera *(the honeybee), and the Tra2-like protein from the jewel wasp *Nasonnia vitripennis *(Hymenoptera), were then compared (see additional File 3). The number of amino acids composing these Tra2 proteins varied: the *C. capitata *and *B. oleae *had 251 amino acids, *M. domestica *232, *L. cuprina *271, *D. melanogaster, D. virilis *and *D. pseudoobscura *264, 315 and 248 respectively, *B. mori *284, *A. mellifera *269, and the *N. vitripennis *Tra2-like protein 307. These differences are due to changes throughout the Tra2 protein except in the RRM domain, which has the same number of amino acids in all these species.

Similarity was higher among the dipteran Tra2 proteins than between these and the lepipdopteran and hymenopteran Tra2 proteins. The greatest degree of similarity was seen between the tephritid *Anastrepha, Ceratitis *and *Bactrocera *Tra2 proteins (83.9 - 86.3%), and between these and those of the *Lucilia *(57.4 - 58.9%), *Musca *(48.3 - 57.7%), *Drosophila *(36.7% - 49.6%) *Apis *(58.7%) and *Bombyx *(48.2%) representatives.

### The gene *tra-2 *is required for sex determination in *Anastrepha*

**Figure 2:**
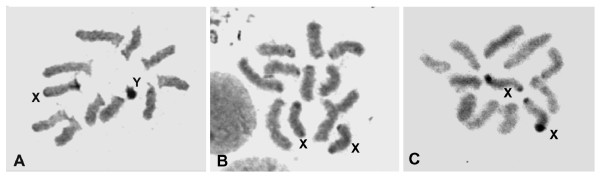
**Chromosomes of *A*. sp.1 *aff. fraterculus***. (A) Normal karyotype in a male that developed from an egg injected with buffer; (B) karyotype of a pseudomale that developed from an egg injected with *tra-2 *dsRNA; and (C) karyotype of a normal female.

Outside *Drosophila*, the function of *tra-2 *in sex determination has been unambiguously demonstrated in *M. domestica *[29] and in *C. capitata *[23] using RNAi procedures. The injection of the *Musca *and *Ceratitis tra-2 *dsRNA into their respective embryos, transformed XX flies into pseudomales. This methodology was used to test the requirement of *tra-2 *for sex determination in *Anastrepha*. An imperative of this technique is to have markers that allow one to determine whether male survivors really do correspond to XX females that have been transformed into pseudomales by destruction of the endogenous *tra-2 *gene function, or normal XY males. No Y chromosome molecular markers have yet been identified for *Anastrepha *flies. Therefore, to ascertain the chromosome constitution of the XX pseudomales, chromosome squashes from the testis of the adults were prepared. The X and the Y chromosome of *A. obliqua *are not easily distinguished [35], whereas those of *A*. sp.1 *aff. fraterculus *can be clearly separated one from another [25] as shown in Figure 2A-C. Hence, embryos of this latter species were used as hosts for the injection of *Aotra2 *dsRNA since the *tra-2 *gene of *A. obliqua *and that of *A*. sp.1 *aff. fraterculus *have a very high degree of similarity (see additional Files 3 and 4) (for details of the injection procedure and the analysis of chromosomes see Materials and Methods).

**Figure 3:**
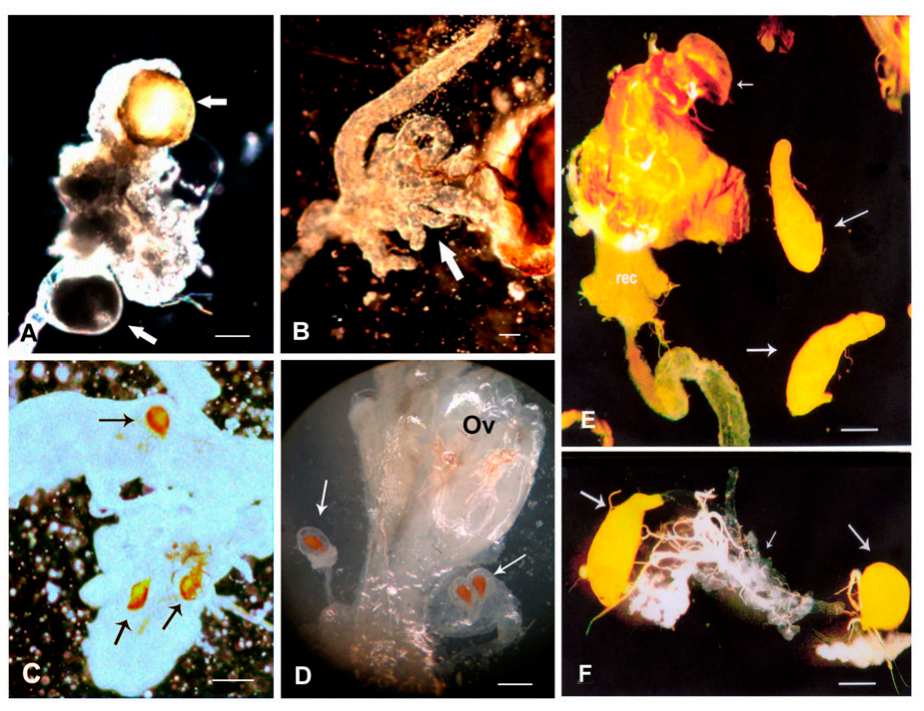
**Internal terminalia of *Anastrepha *flies**. Parts of the reproductive tract of an intersexual mosaic developed from an injected egg with *Aotra2 *dsRNA **(A-C)**, and of a normal female **(D)**. In **(A) **arrows point to two vesicles, one of which is yellow (similar to normal testes). **(B) **Arrow points to the accessory gland. **(C) **Arrows point to the spermathecae. **(D) **Parts of a normal female reproductive tract showing an ovary (ov) and the three regular spermathecae (arrows). Bar = 200 μm. **(E, F) **Parts of the reproductive tract of *A*. sp.1 *aff. fraterculus *males. **(E) **Testes of a normal male (larger arrows). Small arrow points to the male terminalia; rec, rectum. **(F) **Asymmetric testes of a male that developed from an egg injected with *Aotra2 *dsRNA. The right testis is spherical and the other shows the normal elliptical form; the small arrow points to the accessory gland. Bar = 300 μm.

Of the 1450 *A*. sp.1 embryos experimentally injected with *Aotra2 *dsRNA, 86 reached the adult stage, and of these 76 were males and 10 were females. However, among the 1000 control injected embryos, 44 survived to adulthood, 21 being males and 23 females. The sex ratio bias associated with the experimental injection cannot be explained by a higher sensitivity of the female embryos to the injection procedure causing their death. Rather, it suggests a transformation of XX female embryos into XX pseudomales caused by a destruction of the endogenous *tra-2 *function. Testis squashes were prepared for the 76 experimental male survivors; 58 showed cell divisions and the chromosomes were unambiguously identified, showing there to be 47 XY males and 11 XX pseudomales. All these pseudomales showed normal male external terminalia (data not shown). However, after dissection, some of them showed male and female internal genital structures (Figure 3A-C). Others pseudomales had aberrant gonads such as underdeveloped testes, or a well-developed testis plus a poorly developed testis (Figure 3E, F). It was expected that these XX pseudomales would show a change in the splicing pattern of both endogenous *tra *and *dsx *pre-mRNAs. This was confirmed by RT-PCR assays on total RNA from the XX pseudomales, from which the gonads were removed.

**Figure 4:**
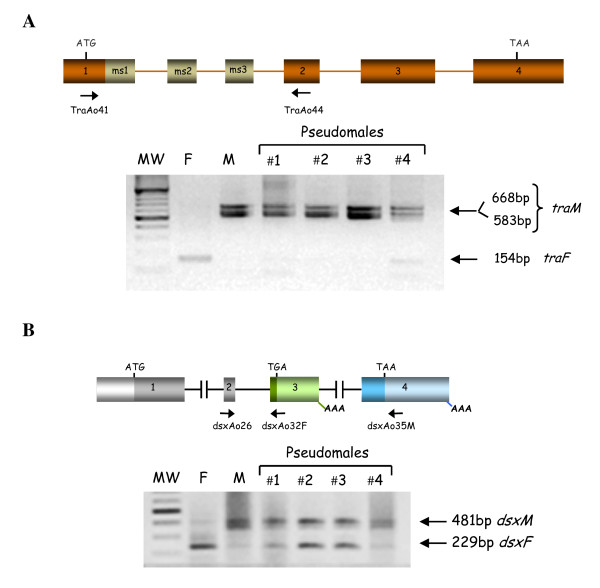
**Analyses of the splicing pattern of *tra *(A) and *dsx *(B) pre-mRNAs of *A. sp.1 aff. fraterculus *in XX pseudomales developed from eggs injected with *Aotra2 *dsRNA**. F and M indicates normal female and male respectively. The sequences of the *TraAo41 *and *TraAo44 *primers used, the locations of which are shown with arrows in Figure 4A, are described in Ruiz et al. [18]; the sequences of the *dsxA26, dsxAo32F *and *dsxAo35M *primers used, the locations of which is shown with arrows in Figure 4B, are described in Ruiz et al. [21].

The gene *tra *of the *Anastrepha *species is transcribed in both sexes during development and in adult life, but its primary transcript follows alternative splicing routes: the male-exons, which are incorporated into mature mRNA in males are spliced out in females. It encodes three female mRNAs that differ in the length of the 3'-UTR depending on the poly-A(+) signal used, and five different isoforms of male mRNA depending on the male-specific exons included [18]. To analyse the splicing pattern of the endogenous *tra *pre-mRNA, the pair of primers TraAo41 and TraAo44 were used; these are located in common exons 1 and 2, respectively, which flank the male-specific exons (Figure 4A). Figure 4A shows that in the pseudomales the amplicons corresponding to the male *tra *mRNA isoform were generated (traces of the female *tra *mRNA were present in pseudomale #4). Negative controls in all these PCR reactions produced no amplicons (see Materials and Methods). These results indicate that the destruction of the endogenous *tra-2 *function causes a change in the splicing pattern of the endogenous *tra *pre-mRNA from the female mode into the male one. Consequently, the Tra2 protein is required for sex determination in *Anastrepha *through its participation in the female-specific splicing of *tra *pre-mRNA.

The gene *dsx *of *Anastrepha *codes for male-and female-specific RNAs, which encode the male-specific DsxM and female-specific DsxF proteins [20,21]. The presence of putative Tra-Tra2 binding sites in the female-specific exon of *dsx *suggests suggest that, as in *Drosophila*, male-specific splicing represents the default mode and that female-specific splicing requires the Tra-Tra2 complex. To analyse the splicing pattern of the endogenous *dsx *pre-mRNA in the XX pseudomales, the pair of primers dsxAo26 in the common exon 2 and dsxAo32F in the female-specific exon were used to detect the female *dsxF *mRNA, whereas the pair of primers dsxAo26 in the common exon 2 and dsxAo35M in the male-specific exon were used to detect the male *dsxM *mRNA (Figure 4B). The four analysed XX pseudomales had the *dsxM *mRNA (Figure 4B), but three of then (#1, 2 and 3) showed also the *dsxF *mRNA although in different abundance. While in pseudomale #1 the transcript was barely visible, in pseudomales #2 and 3, the amount was almost similar relatively to the corresponding *dsxM *RNA. Negative controls in all these PCR reactions produced no amplicons (see Materials and Methods). These results indicate that the Tra2 protein is needed for the female-specific splicing of *dsx *pre-mRNA in *Anastrepha*.

### Phylogeny and molecular evolution of gene *tra-2*

**Figure 5:**
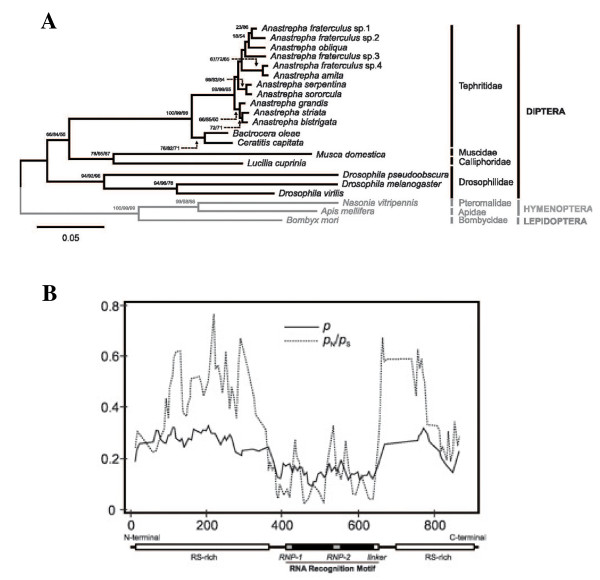
**Molecular evolution of Tra2 proteins**. **(A) **Amino acid phylogeny encompassing Tra2 proteins from Diptera. Taxonomic relationships are indicated in the right margin of the tree. Numbers for interior nodes represent bootstrap and confidence probabilities based on 1000 replicates, followed by the BP corresponding to the maximum parsimony tree topology (shown only when greater than 50%). The topology was rooted with the Tra2 protein from the lepidopteran *B. mori *and the hymenopterans *A. mellifera *and *N. vitripennis*. **(B) **Proportion of nucleotide sites at which two sequences being compared were different (*p*, nucleotide substitutions per site) and ratio between the numbers of non-synonymous (*p*_N_) and synonymous (*p*_S_) substitutions per site across the coding regions of *tra-2 *in the analysed species. The different functional regions defined for the Tra2 proteins are indicated below the graph.

The Tra2 protein sequences determined for different *Anastrepha *species were aligned with homologous sequences from other tephritids and representative insects in order to reconstruct the evolutionary relationships of this protein in Diptera (see additional File 4), using the Tra2 sequences from *B. mori *(Lepidoptera), *A. mellifera *and *N. vitripennis *(Hymenoptera) as outgroups. The topology obtained for the Tra2 protein phylogeny shown in Figure 5A shows high confidence levels in the groups defined. In the phylogenetic tree, the Tra2 proteins of the *Nasonia, Apis *and *Bombix *representatives clustered in a basal clade and the dipteran species in another. Within the latter, *Drosophila *species were found in one branch and the other dipterans in another. *Musca *and *Lucilia *clustered in a subgroup and the species of Tephritidae in another. Among the tephritids, *Bactrocera *and *Ceratitis *clustered into one subgroup on the same branch, while the *Anastrepha *species clustered into a different subgroup of that same branch.

The Tra2 protein from the *Anastrepha *species appears to be closely related to Tra2 from other tephritids, such as *Ceratitis *and *Bactrocera*, as well as to *Musca *and *Lucilia*, showing a monophyletic origin. The Tra2 proteins of the *Drosophila *representatives, however, show substantial divergence.

The variation presented both at the nucleotide and amino acid levels of the *tra-2 *gene in Diptera was studied in the present work by discriminating between the different functional domains of Tra2 protein: the RRM, RS N-terminal (RS1) and RS C-terminal (RS2) domains and the linker region. The overall amino acid and nucleotide variation were 0.236 ± 0.011 and 0.359 ± 0.021 substitutions per site, respectively, are given in additional File 5. The nature of this nucleotide variation was essentially synonymous, being significantly greater than the non-synonymous variation in all cases except for the N-terminal RS1 domain of the protein. The overall variation obtained in Diptera was substantially higher than that seen for the tephritids, which showed a high degree of protein conservation among themselves (0.048 ± 0.007 amino acid substitutions per site and 0.081 ± 0.006 nucleotide substitutions per site). In the tephritids - as for the dipterans - the level of synonymous variation was significantly greater than that of non-synonymous variation. Comparisons between the extent of synonymous and non-synonymous variation revealed significant differences in all cases (*Z*-test, *P *< 0.001; see additional File 5) with the exception of the N-terminal RS1 domain of Tra2, pointing towards the presence of negative selection acting at the protein level in order to maintain Tra2 structural features, especially with respect to the RRM domain.

A higher degree of protein and nucleotide sequence conservation in the Tra2 protein was evident in the region corresponding to the RRM motif as well as in the linker region (adjacent to RRM), in contrast to the high degree of variation presented by the RS1 and RS2 domains. The nucleotide variation across the *tra-2 *gene is detailed in the graph shown in Figure 5B. A valley in the region corresponding to the RRM and linker regions were observed concomitantly with a reduction in non-synonymous substitutions, emphasizing the critical role played by this RRM domain in Tra2 function.

## Discussion

In this work, *Aotra2 *was characterised and found to produce a single mRNA in both sexes that encoded a 249 amino acid-long protein with the features of the SR protein family. In contrast to that seen in *Drosophila*, no significant partially spliced mRNA isoform specific to the male germ line was detected. The observed mRNA is transcribed in both sexes during development and in adult life in both the soma and germ line. The injection of *Aotra-2 *dsRNA into *Anastrepha *embryos caused a change in the splicing pattern of the endogenous *tra *and *dsx *pre-mRNAs of XX females from the female to the male mode. Consequently, these XX females showed transformation into pseudomales.

The recover of XX pseudomales composed of a mixture of male and female structures in the internal genitalia indicates that the transformation of females induced by the *Aotra-2 *dsRNA was partial. This result is in line to observations of partial transformation induced by RNAi of the gene *tra *of *C. capitata *[16], of *B. oleae *[17], of *L. cuprina *[30] and of *M. domestica *[36], and the partial transformation induced by RNAi of the gene *tra-2 *of *M. domestica *[29] and of *C. capitata *[23], in which some pseudomales had, e.g. male external genitalia but female structures in the anterior regions of the body. Unfortunately, in *Anastrepha *species, including the *A. sp.1 *here studied, the degree of sexual mosaicism at the morphological level cannot be inferred since there are no sexually dimorphic structures in the adult body other than the genitalia (external and internal) and the external analia [37]. Based on the results found in *C. capitata, B. oleae, L. cuprina *and *M. domestica *above mentioned, it is expected that in *A. sp.1 *the extent of the sexual transformation was also variable among the pseudomales; i.e. although these were identified as males by inspection of the external terminalia, other structures of the fly could remain female if the injected dsRNA did not reach their cellular precursors. This would explain the fact that the analysed *A. sp.1 *XX pseudomales showed *traF *and *dsxF *transcripts besides the expected *traM *and *dsxM *mRNAs. The variable amount of the female transcripts found in the pseudomales would be related to the different proportions of female structures in their non-sexually dimorphic regions. These results indicate that the Tra2 protein is needed for sex determination in *Anastrepha*. 

Finally, the Tra2 proteins of ten other *Anastrepha *species were characterised and compared among themselves and with the Tra2 protein of other insects in which it has been characterised. The Tra2 protein from the *Anastrepha *species was closely related to the Tra2 from other tephritids, such as *Ceratitis *and *Bactrocera*, as well as to other dipterans such as *Musca *and *Lucilia*, showing a monophyletic origin. However, the Tra2 protein of *Drosophila *showed substantial divergence. The nature of the nucleotide variation in *tra-2 *was essentially synonymous (significantly more common than non-synonymous variation). This suggests the existence of negative selection acting at the protein level in order to maintain Tra2 structural features, especially with respect to the RRM domain.

### Function of *tra-2 *in *Anastrepha *sex determination

In the tephritids *C. capitata *[16] and *B. oleae *[17], and in twelve *Anastrepha *species [18], the gene *tra *acts as the memory device for sex determination via its auto-regulatory function, i.e., through the contribution of the Tra protein to the female-specific splicing of its own pre-mRNA [16,17]. Further, the *tra-2 *of *C. capitata *is needed for the female-specific splicing of *tra *and *dsx *pre-mRNAs [23]. This requires the formation of a complex with the Tra protein, which then interacts with the Tra-Tra2 binding sites present in both pre-mRNAs [16,19]. This role for *tra-2 *in sex determination also exists in *Anastrepha *species (present work).

**Figure 6:**
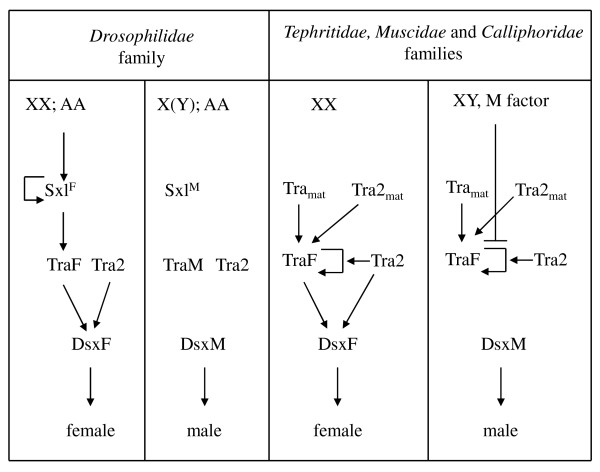
**Comparison of the sex determination gene cascades between *Drosophilidae (Drosophila)*, and *Tephritidae (Ceratitis Anastrepha *and *Bactrocera), Muscidae (Musca) *and *Calliphoridae (Lucilia) *families of Diptera**. Tra_mat _and Tra2_mat _indicate maternal Tra and Tra2 product. DsxF and DsxM stands for female and male Dsx protein, respectively. Though the maternal expression of gene *tra-2 *in *Lucilia *and the characterisation of this gene in *Bactrocera *have not been reported, both species are included in the scheme of this Figure because the analysis of *tra *in *Bactrocera *[17] and the analysis of *tra *and *tra-2 *in *Lucilia *[30] suggests their *tra-2 *genes have a similar function as in *Ceratitis, Anastrepha *and *Musca*. Scheme based on references 16, 17, 18, 23, 29, 30 and 36.

The maternal expression of *tra *[16-18] and *tra-2 *([23]; present work) in tephritids supplies the embryo with maternal Tra-Tra2 complex. This is essential for imposing female-specific splicing of the initial zygotic *tra *pre-mRNA so that the first zygotic functional Tra protein is produced and *tra *auto-regulation can be established. In this scenario the XX embryos follow female development. In XY embryos, however, the yet non-characterised M factor present in the Y chromosome would prevent the *tra *auto-regulation system being set up. Consequently, these embryos would not produce functional Tra protein and develop as males [16] (see Figure 6). The existence of an M factor in the Y chromosome has been demonstrated only in *C. capitata *[38]. However, the analysis of *tra *in *Bactrocera *[17] and *tra *[18] and *tra-2 *(present work) in *Anastrepha *species suggests their Y chromosome have a similar function.

Both the tephritid Tra and Tra2 protein show a dual splicing role in sex determination. On one hand both behave as a splicing activator of *dsx *pre-mRNA: the binding of Tra-Tra2 to the female-specific exon promotes the inclusion of this exon into the mature mRNA. On the other hand, Tra and Tra2 act as splicing inhibitors of *tra *pre-mRNA: the binding of Tra-Tra2 to the male-specific exons prevents the inclusion of these exons into the mature mRNA. It has been proposed that the Tra2-ISS binding sites, which have been found in the splicing regulatory region of the *tra *pre-mRNA of the tephritids, but not in *dsx *pre-mRNA, provide the distinguishing marker for the dual splicing function of the Tra-Tra2 complex in tephritids [18].

This role of genes *tra *and *tra-2 *in sex determination is not exclusive to tephritid insects (Diptera, Tephritidae) since the homologous genes in *L. cuprina *(Diptera, Calliphoridae) seem to play the same role [30]. A similar situation is found in the housefly *M. domestica *(Diptera, Muscidae), where the gene *F *plays the key role for female sex determination. The maternal product is needed to activate the zygotic function of *F*, which appears to show auto-regulation [39]. Recently, the molecular characterisation of *F *revealed it to be the orthologue of *tra *in the housefly [36]. *tra-2 *is also required for this auto-regulation in this species [29]. The existence of an M factor in the Y chromosome has been also demonstrated in *Lucilia *[40] and in *Musca *[41-43], though in this latter species, some strains carry the M factor in an autosome [44]. In XY zygotes, the presence of the masculinising factor M in the Y chromosome would prevent the establishment of *tra *auto-regulation and cause male development [30,36] (see Figure 6).

Together, these results support the model of Wilkins [45], who proposed that the evolution of sex-determining cascades was bottom up (for a theoretical analysis of this model see Pomiankowski et al., [46]). It has been suggested [18] that the *tra/tra2 > dsx *elements at the bottom of the cascade, and their relationships, likely represent the ancestral state (which still exists in the Tephritidae, Calliphoridae and Muscidae lineages) of the extant cascade found in the Drosophilidae lineage (in which *tra *is just another component of the sex determination gene cascade regulated by *Sxl*). Thus, in the phylogenetic lineage that gave rise to the drosophilids, evolution co-opted for the *Sxl *gene, modified it, and converted it into the key gene controlling sex determination.

In *Drosophila*, the gene *tra-2 *also shows a dual splicing role. It behaves as a splicing activator of *dsx *pre-mRNA in the soma of *Drosophila *females, but also acts as a splicing inhibitor of the M1 intron in *tra-2 *pre-mRNA in the germ line of *Drosophila *males (see Figure 1C). The presence of M1 in the mature *tra-2 *mRNA prevents the formation of full, functional Tra2 protein [32]. It has been found that *Drosophila *somatic cells are also able to prevent splicing of the M1 intron whenever levels of Tra2 protein are above normal [47]. The *tra-2 *promoter of the male-germ line is more active than the *tra-2 *promoter of the somatic tissues of *Drosophila *[27,28,47]. Regulated levels of Tra2 protein are therefore required since an excess of the Tra2 protein causes male sterility [33] and reduces the viability of *Drosophila *males and females [47].

The gene *tra-2 *produces a single mRNA in both sexes and no significant partially spliced mRNA isoform specific to the male germ line has been detected in *Ceratitis *[23], *Musca *[29], *Lucilia *[30] or *Anastrepha *(present work). Hence, it is here postulated that the promoter of this gene in the male germ line of these dipteran insects behaves in a fashion similar to that seen in the somatic tissues. The splicing inhibitor function of *tra-2 *observed in the *Drosophila *male germ line was probably acquired during the evolutionary lineage that gave rise to the drosophilids and may constitute an evolutionary solution for its high expression in this tissue and the return to normal levels of Tra2 protein. This protein belongs to the SR protein family, the members of which are involved in splicing regulation, mRNA transport and mRNA translation [48]. Its level in the cell must therefore be regulated if these processes are not to be impaired.

### The molecular evolution of Tra2

The analysis of Tra2 relationships among the studied species resulted in a statistically well supported phylogenetic tree. In this tree, the Tephritidae species, which belong to the subgroup Acalyptratae of the suborder Brachycera, were more closely related to *Musca *and *Lucilia*, which belong to the Calyptratae subgroup of the suborder Brachycera, than to *Drosophila *species, which also belong to the subgroup Acalyptratae. These findings are in line with previous analyses of genes involved in sex determination, e.g., *Sxl *[49], *tra *[30] and *tra-2 *[22,30]. However, the analysis of *dsx *indicates a distinct relationship, i.e., Dsx proteins from the Tephritidae are more closely related to *Drosophila *than to *Musca *[21]. This discrepancy might be explained by the position of the genes *dsx, tra *and *tra-2 *in the sex determination cascade. The gene *dsx *is the more basal gene in this cascade, whereas the other genes would have been co-opted for sex determination over evolutionary time; thus, a higher degree of conservation can be expected for *dsx *than for *tra *or *tra-2 *[45]. The gene *dsx *encodes for transcription factors that control the sexual cytodifferentiation genes; *dsx *is therefore subject to strong purifying selection to maintain this control. The Tra and Tra2 proteins belong to the SR family of proteins involved in splicing, which are characterised by having repetitions of the serine-arginine dipeptide. Variation in the content of RS dipeptides seems to be a feature of the SR proteins whenever they have maintained enough such dipeptides to preserve their function [50].

The overall amino acid variation of Tra2 (present work) is significantly smaller than that previously reported for Tra [18]. The low rates of evolution shown by Tra2 in tephritids are also in agreement with the low rates of evolution reported for Tra in this group, and in stark contrast with the high rates of neutral evolution reported in some *Drosophila *species [50,51].

The extent of synonymous and non-synonymous nucleotide variation in the Tra2 proteins suggests the presence of extensive silent divergence. This, together with the strict conservation of the distribution of the RS-rich and RRM domains, suggests that *tra-2 *is subject to strong purifying selection to preserve the mechanism of action of Tra2 proteins. A higher degree of conservation was evident in the RRM and linker regions, in contrast to the diversity shown by the RS-rich regions. In fact, the RRM-linker junction region is considered a signature motif of Tra2 proteins [52].

The Tra protein seems to lack an RNA binding domain; thus, its influence in splicing regulation is exerted at the level of its interaction (through the RS domains) with other proteins carrying RNA-binding domains, such as Tra2 (reviewed in [53]). The variation in the number of RS dipeptides in the RS1 and RS2 regions of the Tra2 proteins parallels the situation found in the Tra proteins, which appear to undergo high rates of neutral evolution [50,51]. The high degree of conservation in the RRM domain of the Tra2 proteins studied here agrees with its fundamental role in the function of the Tra-Tra2 complex; this domain confers upon the complex its capacity to specifically interact with the *tra *and *dsx *pre-mRNAs and thus regulate its sex-specific splicing.

The high degree of divergence between the *Anastrepha *and the *Drosophila *Tra2 proteins is of particular interest. This divergence was mainly observed in the RS domains, which are involved in protein-protein interactions. This observation agrees with the experimental observation that the *Anastrepha *Tra-*Drosophila *Tra2 complex appears to be less efficient than the *Drosophila *Tra-Tra2 complex at inducing female-specific splicing of the *Drosophila dsx *pre-mRNA [54]. Hence, the interaction between the *Anastrepha *Tra protein and the *Drosophila *Tra2 protein might be impeded as a consequence of changes accumulated in these proteins after the *Anastrepha *and *Drosophila *phylogenetic lineages separated. These results suggest that Tra and Tra2 proteins co-evolved to exert their function in sex determination.

## Conclusions

The gene *transformer-2 *is required for sex determination in *Anastrepha *through its participation in the female-specific splicing of *transformer *and *doublesex *pre-mRNAs. It is therefore needed for the auto-regulation of the gene *transformer*. Thus, the *transformer/transfomer-2 > doublesex *elements at the bottom of the cascade, and their relationships, probably represent the ancestral state (which still exists in the Tephritidae, Calliphoridae and Muscidae lineages) of the extant cascade found in the Drosophilidae lineage (in which *tra *is just another component of the sex determination gene cascade regulated by *Sex-lethal*). The extent of synonymous and non-synonymous nucleotide variation in the Tra2 proteins suggests the presence of extensive silent divergence. This, together with the strict conservation of the distribution of the RS-rich and RRM domains, suggests that *tra-2 *is subject to strong purifying selection to preserve the mechanism of action of Tra2 proteins.

## Methods

### Species

The species of *Anastrepha *studied, their host fruits, and the sites where they were collected are described in Ruiz et al. [21]. Rearing conditions were 23 ± 2°C, relative humidity 60-80%, light intensity 4000-5000 lux, photoperiod 12:12 h (L:D).

### Molecular analyses

Genomic DNA was extracted from frozen specimens as described in Maniatis et al. [55]. Total RNA from adult female ovaries, adult male testis, embryos, larvae, and adult male and female somatic cells was prepared using the Ultraspec-II RNA isolation kit (Biotecx) following the manufacturer's instructions. Five micrograms of total RNA from each sample were reversed transcribed with Superscript (Invitrogen) following the manufacturer's instructions. Reverse transcription reactions were performed with an oligo-dT. Two percent of the synthesised cDNA was amplified by PCR. All amplicons were analysed by electrophoresis in agarose gels, cloned using the TOPO TA-cloning kit (Invitrogen) following the manufacturer's instructions and sequenced. In all cases, PCR reactions with RNA samples were performed to guarantee that they were not contaminated with genomic DNA (negative controls of PCR reactions).

The GenomeWalker genomic library of *A. obliqua *was synthesised using the BD GenomeWalker Universal kit (BD Biosciences), following the manufacturer's instructions.

Southern-blotting (see Figure 1F) was performed by transferring the RT-PCR products shown in Figure 1E onto a nylon membrane (Zeta-Probe Blotting Membranes BIO RAD). The probe used corresponds to a fragment of 630 nucleotides of the *A. obliqua tra-2 *gene, obtained by PCR involving genomic DNA and using the pair of primers PM1 and PM2 located at the 5' and 3' ends of intron 3 respectively (see Figure 1A). The probe was labelled with digoxigenin using the PCR DIG Labeling Mix kit (Roche) and hybridisation was detected with the DIG luminescent Detection Kit for Nucleic Acids (Roche) following the manufacturer's instructions.

### DNA sequencing and sequence analysis

Sequencing was performed using an automated 377 DNA sequencer (Applied Biosystems). The analysis of sequences was performed by using the BLASTX programme.

### Injection of *Aotra2 *dsRNA into *Anastrepha *embryos

The *tra2 *dsRNA was prepared as described for *Drosophila *[56]. The complete ORF of *Aotra2 *cloned in the pUAST plasmid was used as a template in a PCR reaction with the primer pair P1T7 and P3T3 (corresponding to primers P1 and P3) (see Figure 1A), flanked by a T7 promoter sequences at their 5' ends. The amplicon of this PCR was used as template to produce the *tra2 *dsRNA in an *in vitro *transcription reaction with T7 RNA polymerase using the Megascript kit (Ambion). This dsRNA was precipitated with ethanol and resuspended in the injection buffer [57].

Hemispheres made of 3% agar stained with red commercial food dye (aniline) and wrapped in parafilm^® ^where furnished for oviposition [25], and eggs for injection recovered after 2 h. These were injected into the adjacent anterior region of the posterior pole with either 8 μM *Aotra2 *dsRNA (experimental embryos) or injection buffer (control embryos), following the described procedures [56] with minor modifications. The injected embryos were then transplanted into host fruit where they developed until reaching the pupal stage. The pupae were collected and transferred to population cages until the emergence of the adults. Newborn males (2-4 days old) were examined to record their external terminalia (external genitalia plus analia), which is the only external sexual dimorphic structure found in *Anastrepha *species [37], and then dissected for analysis of the internal genital structures and the removal of the testes for the determination of their chromosomal constitution.

### Preparation of chromosome squashes

Chromosome squashes of testes from the *Anastrepha *males were prepared as previously described [58,59].

### Sequences of the primers used in this work

Mar17: 5' GTRTGIGSIVGYTKNGTDATNGA 3';

Mar26: 5' MGIAGYCGNGGNTTYTGYTTY 3';

Tra2B: 5' NCKRTCRTCDATYTCCATNCC 3';

P1: 5' AGAGTTGGAATGAGTCCACG 3';

P2: 5' CACGTCGCTTATCGTATGGA 3';

P3: 5' CATATTTTTAATAGCGCGTACG 3';

P4: 5' ATTACCAAGGTGTGGGCTTC 3';

P5: 5' AGTGAAATCCAGTTGATACGC 3';

GW1: 5' TATCAGGATATAGCCGATGCTAAGGC 3';

GW2: 5' CAAGCGTCTTTAGCTGCCTTAGCATC 3';

PM1: 5' TACGAACGCAGCTTACTTCC 3';

PM2: 5' CTTGCGGTTCTGAGACTGAC 3'.

P1T7: 5' TAATACGACTCACTATAGGGACTAGAGTTGGAATGAGTCCACG 3'

P3T3: 5' TAATACGACTCACTATAGGGACTCATATTTTTAATAGCGCGTACG 3'

The Mar17 primer is described in Burghardt et al. [29]. The sequence of the Mar26 and Tra2B primers was generously provided by K. Komitopoulou.

### Phylogenetic and Molecular Evolutionary Analyses

The present analysis included 21 *tra-2 *sequences belonging to different insects with the following accession numbers: DIPTERA: *Anastrepha amita *[EMBL: FN658617], *A. bistrigata *[EMBL: FN658616], *A. fraterculus *sp.1 [EMBL: FN658608], *A. fraterculus *sp.2 [EMBL: FN658609], *A. fraterculus *sp.3 [EMBL: FN658610], *A. fraterculus *sp.4 [EMBL:FN658611], *A. grandis *[EMBL:FN658612], *A. obliqua *[EMB:FN658607], *A. serpentina *[EMBL: FN658613], *A. sororcula *[EMBL: FN658614], *A. striata *[EMBL: FN658615], *Ceratitis capitata *[EMBL:EU999754], *Bactrocera oleae *[EMBL:AJ547623], *Musca domestica *[EMBL:AY847518], *Lucilia cuprinia *[EMBL:FJ461620], *Drosophila melanogaster *[EMBL:M23633], *D. virilis *[EMBL:XM_002049663], *D. pseudoobscura *[EMBL:XM_001360568]; LEPIDOPTERA: *Bombyx mori *[EMBL:NM_001126233]; HYMENOPTERA: *Apis mellifera *[EMBL:XM_001121070] and *Nasonia vitripennis *[EMBL:XP_001601106)]. Multiple sequence alignments were conducted on the basis of the translated amino acid sequences and edited for potential errors over 1089 nucleotide sites corresponding to 363 amino acid positions using BIOEDIT [60] and CLUSTAL W [61] software. The different domains in the Tra2 protein were defined according to Salvemini et al. [23] as: the RS-rich N-terminal region (19-459), the RNA recognition motif-RRM (502-717), the linker region (718-774), and the RS-rich C-terminal region (775-1077).

Molecular evolutionary analyses were performed using MEGA v.4 software [62]. The extent of nucleotide sequence divergence was estimated by means of the Kimura 2-parameter method [63] and the amino acid sequence divergence estimated by means of the uncorrected differences (*p*-distances). This approach is known to give good results, especially for distantly related taxa [64]. The number of synonymous (*p*_S_) and non-synonymous (*p*_N_) nucleotide differences per site were calculated using the modified method of Nei-Gojobori [65], providing the transition/transversion ratio (R) for each case. Evolutionary distances were calculated using the compete deletion option. Standard errors of the estimates were calculated using the bootstrap method (1000 replicates). Phylogenetic trees were reconstructed using the neighbour-joining tree-building method [66]. However, maximum parsimony trees were also inferred to ensure that the obtained results were not dependent on this choice. The reliability of the resulting topologies was tested by the bootstrap method [67] and by the interior-branch test [68], producing the bootstrap probability (BP) and confidence probability (CP) values for each interior branch in the tree respectively. Given that the bootstrap method is known to be conservative, a BP > 80% was interpreted as high statistical support for groups, whereas a CP ≥ 95% was considered statistically significant [69]. Phylogenies were rooted using the *tra-2 *gene of Lepidoptera and Hymenoptera.

The analysis of the nucleotide variation across coding regions was performed using the sliding-window approach afforded by DnaSP v.5 software [70], estimating the proportion (*p*) of nucleotide sites at which two sequences being compared were different, and the ratio between the numbers of non-synonymous (*p*_N_) and synonymous (*p*_S_) substitutions per site, employing a window length of 25 nucleotides and a step size of 5 nucleotides.

## Authors' contributions

FS performed the experiments. MFR helped with the molecular biology experiments. JMEL performed the phylogenetic analyses. ALPP performed the analysis of chromosomes. DS performed the analysis of chromosomes and supervised the dsRNA injection experiments. LS conceived and supervised the study, and wrote the manuscript. All authors contributed to the final version of the manuscript.

## Supplementary Material

Additional file 1 **Number of nucleotides that compose the exons and introns of gene *tra-2 *of *A. obliqua, C. capitata *and *D. melanogaster***. Exons (boxes) and introns (lines) are not drawn to scale. Click here for file

Additional file 2 **Percentage similarity among the *Anastrepha *Tra2 proteins**. Comparison of the Tra2 proteins of *Anastrepha *species. Click here for file

Additional file 3 **Percentage similarity among the insect Tra2 proteins**. Comparison of the Tra2 proteins of the insects so far characterised. *A. obliqua *was used as the reference species for the *Anastrepha *species. Click here for file

Additional file 4 **Amino acid alignment of the Tra2 protein in the analysed species**. The different protein domains are indicated as follows: RS-rich regions in grey background, RNA recognition motif (RRM) in green background, and linker region in yellow background. The RNP-1 and RNP-2 elements are indicated in open black boxes. Click here for file

Additional file 5 **Evolutionary parameters of the Tra2 proteins from insects**. Average numbers of amino acid (*p*_AA_), total nucleotide (*p*_*NT*_), synonymous (*p*_S_) and non-synonymous (*p*_N_) nucleotide differences per site, codon bias (Effective Number of Codons, ENC) and *Z*-test of selection in complete *tra-2 *genes and independent *tra-2 *domains from insects. SE, standard error; R, average transition/transversion ratio; n/a, not applicable. ^a ^H_0_: *p*_N _= *p*_S_; H_1_: *p*_N _<*p*_S_. Click here for file
