# Supplementary material for: The gene transformer-2 of Anastrepha fruit flies (Diptera, Tephritidae) and its evolution in insects
Source: BMC Evol Biol. 2010 May 13;10:140. doi: 10.1186/1471-2148-10-140 (PMC2885393; doi:10.1186/1471-2148-10-140)
Supplement: Additional file 1 — Number of nucleotides that compose the exons and introns of gene tra-2 of A. obliqua, C. capitata and D. melanogaster. Exons (boxes) and introns (lines) are not drawn to scale. [file 1471-2148-10-140-S1.PPT]

## Slide 1
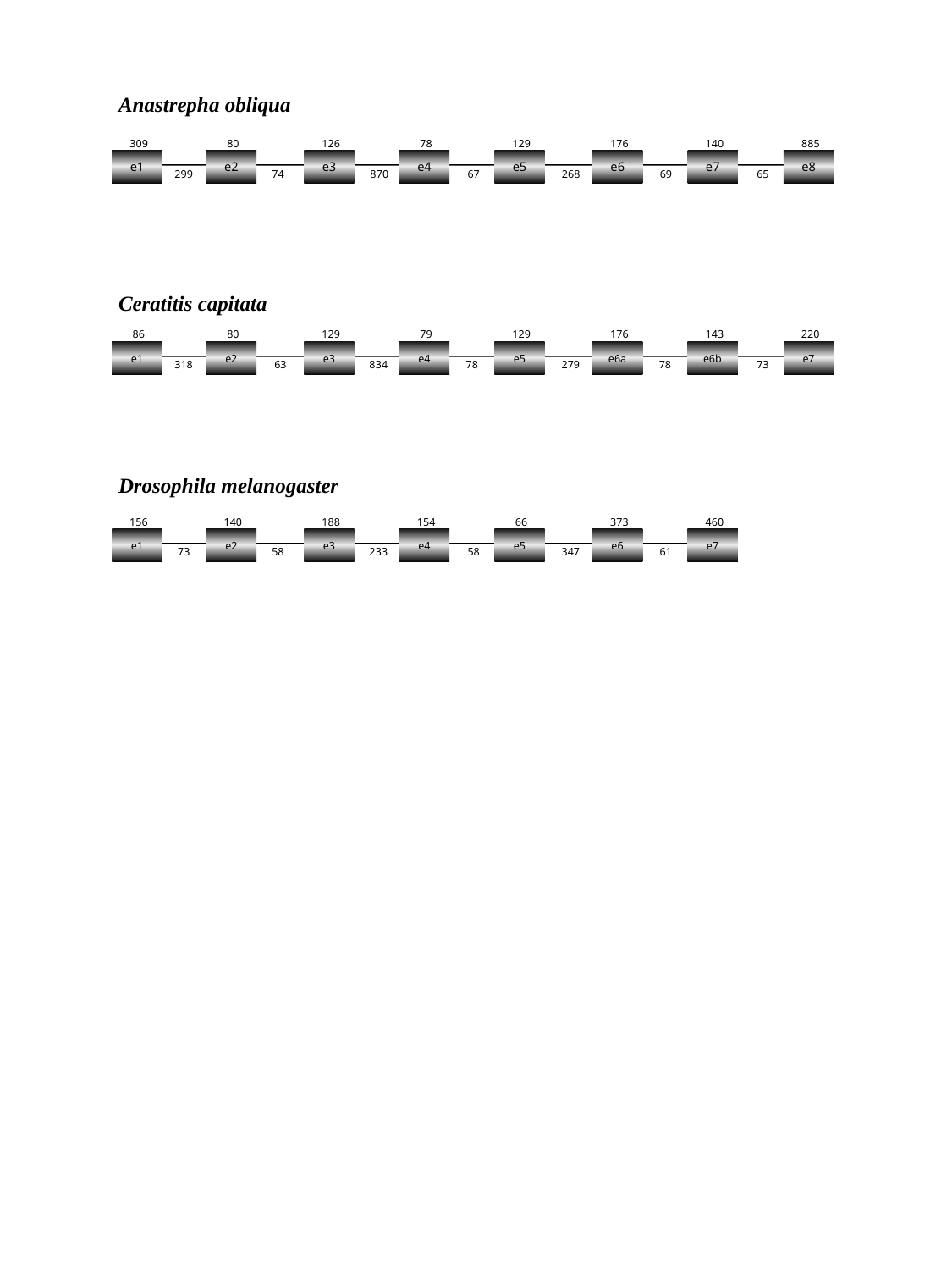

Anastrepha obliqua
309
80
126
78
129
176
140
885
e1
e2
e3
e4
e5
e6
e7
e8
299
74
870
67
268
69
65
Ceratitis capitata
86
80
129
79
129
176
143
220
e1
e2
e3
e4
e5
e6a
e6b
e7
318
63
834
78
279
78
73
Drosophila melanogaster
156
140
188
154
66
373
460
e1
e2
e3
e4
e5
e6
e7
73
58
233
58
347
61
