# Supplementary material for: The gene transformer-2 of Anastrepha fruit flies (Diptera, Tephritidae) and its evolution in insects
Source: BMC Evol Biol. 2010 May 13;10:140. doi: 10.1186/1471-2148-10-140 (PMC2885393; doi:10.1186/1471-2148-10-140)
Supplement: Additional file 2 — Percentage similarity among the Anastrepha Tra2 proteins. Comparison of the Tra2 proteins of Anastrepha species. [file 1471-2148-10-140-S2.DOC]

**Table 1: Percentage similarity among the *Anastrepha* Tra2 proteins.**

| ***Anastrepha*** | ***fsp1*** | ***fsp2*** | ***fsp3*** | ***fsp4*** | ***grandis*** | ***serpentina*** | ***sororcula*** | ***striata*** | ***bistrigata*** | ***amita*** |
| --- | --- | --- | --- | --- | --- | --- | --- | --- | --- | --- |
| ***obliqua*** | 98.8 | 97.9 | 98.4 | 98.4 | 97.6 | 97.2 | 97.5 | 97.9 | 97.6 | 98.4 |
| ***fsp1*** |  | 99.2 | 99.6 | 99.6 | 98.8 | 98.4 | 98.8 | 99.2 | 98.8 | 99.6 |
| ***fsp2*** |  |  | 98.8 | 98.8 | 97.9 | 97.6 | 97.9 | 98.4 | 97.9 | 98.8 |
| ***fsp3*** |  |  |  | 99.2 | 98.4 | 97.9 | 98.4 | 98.8 | 98.4 | 99.2 |
| ***fsp4*** |  |  |  |  | 98.4 | 97.9 | 98.4 | 98.8 | 98.4 | 100 |
| ***grandis*** |  |  |  |  |  | 97.2 | 97.6 | 98.8 | 98.4 | 98.4 |
| ***serpentina*** |  |  |  |  |  |  | 99.6 | 97.6 | 97.2 | 97.9 |
| ***sororcula*** |  |  |  |  |  |  |  | 97.9 | 97.6 | 98.4 |
| ***striata*** |  |  |  |  |  |  |  |  | 99.2 | 98.8 |
| ***bistrigata*** |  |  |  |  |  |  |  |  |  | 98.4 |
