# Supplementary material for: The gene transformer-2 of Anastrepha fruit flies (Diptera, Tephritidae) and its evolution in insects
Source: BMC Evol Biol. 2010 May 13;10:140. doi: 10.1186/1471-2148-10-140 (PMC2885393; doi:10.1186/1471-2148-10-140)
Supplement: Additional file 3 — Percentage similarity among the insect Tra2 proteins. Comparison of the Tra2 proteins of the insects so far characterised. A. obliqua was used as the reference species for the Anastrepha species. [file 1471-2148-10-140-S3.DOC]

**Table 2: Percentage similarity among the Tra2 proteins.**

|  | ***Ceratitis*** | ***Bactrocera*** | ***Lucilia*** | ***Musca*** | ***D.melano*** | ***D.virilis*** | ***D.pseudoobs*** | ***Bombyx*** | ***Apis*** | ***Nasonia*** |
| --- | --- | --- | --- | --- | --- | --- | --- | --- | --- | --- |
| ***Anatrepha*** | 86.3 | 83.9 | 57.4 | 52.1 | 40.6 | 42.1 | 41.1 | 40.6 | 41.4 | 39.3 |
| ***Ceratitis*** |  | 86.4 | 59.8 | 48.3 | 40.6 | 44.2 | 41.5 | 43.4 | 45.4 | 40.6 |
| ***Bactrocera*** |  |  | 58.9 | 51.3 | 41.4 | 44.6 | 41.9 | 42.6 | 42.2 | 41.8 |
| ***Lucilia*** |  |  |  | 57.7 | 36.7 | 39.4 | 39.5 | 40.2 | 41.6 | 36.9 |
| ***Musca*** |  |  |  |  | 41.8 | 41.8 | 40.1 | 41.4 | 46.9 | 43.9 |
| ***D.melano*** |  |  |  |  |  | 51.5 | 49.6 | 35.6 | 37.5 | 36.3 |
| ***D.virilis*** |  |  |  |  |  |  | 46.8 | 35.6 | 36.4 | 32.9 |
| ***D.pseudoobs*** |  |  |  |  |  |  |  | 36.7 | 37.1 | 37.9 |
| ***Bombyx*** |  |  |  |  |  |  |  |  | 50.9 | 48.2 |
| ***Apis*** |  |  |  |  |  |  |  |  |  | 58.7 |
