# Supplementary material for: The gene transformer-2 of Anastrepha fruit flies (Diptera, Tephritidae) and its evolution in insects
Source: BMC Evol Biol. 2010 May 13;10:140. doi: 10.1186/1471-2148-10-140 (PMC2885393; doi:10.1186/1471-2148-10-140)
Supplement: Additional file 4 — Amino acid alignment of the Tra2 protein in the analysed species. The different protein domains are indicated as follows: RS-rich regions in grey background, RNA recognition motif (RRM) in green background, and linker region in yellow background. The RNP-1 and RNP-2 elements are indicated in open black boxes. [file 1471-2148-10-140-S4.DOC]

10 20 30 40 50 60 70

....|....|....|....|....|....|....|....|....|....|....|....|....|....|

**A.obliqua**  **---MSPRT---------RSRSISPR-----------------RSYSKSPARRSNGRRRHSREKVYNSRSR**

**A.fraterculus.sp1** **---.....---------........-----------------............................**

**A.fraterculus.sp2** **---.....---------........-----------------............................**

**A.fraterculus.sp3** **---.....---------........-----------------............................**

**A.fraterculus.sp4** **---.....---------........-----------------............................**

**A.grandis**  **---.....---------........-----------------............................**

**A.serpentina**  **---.....---------........-----------------............................**

**A.sororcula**  **---.....---------........-----------------............................**

**A.striata**  **---.....---------........-----------------............................**

**A.bistrigata**  **---.....---------........-----------------............................**

**A.amita**  **---.....---------........-----------------............................**

**B.oleae**  **---.....---------........-----------------...T...V..G..........F.S....**

**C.capitata**  **---.....---------......A.-----------------...T...A..S..........V.K....**

**M.domestica**  **---.G..S---------....---------------------...S--PED.R....SY..DR-YS.P-V**

**L.cuprinia**  **---.S..S---------H...VTP.-----------------...SRSPYR.S...KSY..YK-YD.RSS**

**D.melanogaster**  **MDREPL-----------SSG.LHCS--------------------ARYKHKRSASSSSAGTTSSGHKDRR**

**D.virilis**  **----------------------------------------------------------------------**

**D.pseudoobscura**  **----------------------------------------------MIKFHSNTKS-------------R**

**B.mori**  **---MSDRE---------RSRSRTRNGSREPVPKPAVMSRGHSRSRSRTPPPPKATSRKYRSPMLTSGLTV**

**A.mellifera**  **---MSDIE---------RSSSRSAS-PRRPRTADGGLRDSRSHSRSRKSRERKESHRPVKEYSRSRSRSV**

**N.vitripennis**  **---MDDVKHHLLPYTQKRSGSRSAS-PRRSR-ADDDMQESRSHSKSSKYREHKEGHRAMKNYSRSRSHSV**

80 90 100 110 120 130 140

....|....|....|....|....|....|....|....|....|....|....|....|....|....|

**A.obliqua**  **SASRHP---------PSPPPLPAGRAG-RYSDASKSSSTPLSPRHGRR-----------------VSRSR**

**A.fraterculus.sp1** ......**---------............-...........S........-----------------.....**

**A.fraterculus.sp2** ......**---------............-...........S........-----------------.....**

**A.fraterculus.sp3** ......**---------............-...........S........-----------------.....**

**A.fraterculus.sp4** ......**---------............-...........S........-----------------.....**

**A.grandis**  ......**---------...........A-...........S........-----------------.....**

**A.serpentina**  .**V....---------L..........G-...........S........-----------------A....**

**A.sororcula**  .**V....---------P..........G-...........S........-----------------A....**

**A.striata**  .**A....---------P..........G-...........S........-----------------V....**

**A.bistrigata**  .**A....---------P...L......G-...........S........-----------------V....**

**A.amita**  .**A....---------P...P......G-...........S........-----------------V....**

**B.oleae**  .**A..Q.---------P...P..S..GG-...E..Q....S........-----------------IT...**

**C.capitata**  .**I..H.---------P...PP.T..GGV.C.D..Q....S....Q...-----------------MS...**

**M.domestica**  .**KR.Q.---------PS..PL.PS.RRS.SRSE.PGYNRRNYSSH.N.-----------------SKPP.**

**L.cuprinia**  .**NS.H.---------PS..PI.TG.HSG.YSSD.RSVSRSVSPTYKKRQHNSRRHYSRSRSRTKSPTRS.**

**D.melanogaster**  .**DYDYC----------GSRRHQRSSSRRRSRSRSSSES---PPPEPRH-------------------RSG**

**D.virilis**  **----------------------------------------------------------------------**

**D.pseudoobscura**  **SHLEYY----------GSNR--SSGSRRRSRSRSYSQSRSISPSGARN-------------------RSR**

**B.mori**  **DGRTHS---------RSRSRSGSARRGYR-SRHSRTRSRSYS-------------------PRGSYRRSH**

**A.mellifera**  **SRGRN----------RSRSRS-RSRSTHRFARYSRSRSRSYFRSRYSRE-----------CDRTIY-RSH**

**N.vitripennis**  **TRSRKSYRSSKYEGRRGGSRS-RSRSPYR-GRASRSRSRSYSRSRYSR-------------DRNMYTRSH**

150 160 170 180 190 200 210

....|....|....|....|....|....|....|....|....|....|....|....|....|....|

**A.obliqua**  **SRSPYDKR-RANREK------PVQNRCIGVFGLSVYTTQQKIRDIFSRFGPIERIQVVIDAQTGRSRGFC**

**A.fraterculus.sp1** **T.......-......------.................................................**

**A.fraterculus.sp2** **T.......-......------.................................................**

**A.fraterculus.sp3** **T.......-......------.................................................**

**A.fraterculus.sp4** **T.......-......------.................................................**

**A.grandis**  **T.......-......------.................................................**

**A.serpentina**  **T.......-......------.................................................**

**A.sororcula**  **T.......-......------.................................................**

**A.striata**  **T.......-....K.------.................................................**

**A.bistrigata**  **T.......-....Q.------.................................................**

**A.amita**  **T.......-....E.------.................................................**

**B.oleae**  **S..THEN.-.G..E.------.................................................**

**C.capitata**  **S..PYDK.-.G..E.------.................................................**

**M.domestica**  **S..PYDRNY.N..E.------.SPC..L.......H....Q..E...KY.........V...........**

**L.cuprinia**  **S.NSYDRAN.S..E.------.LPC..I.......Y...LK..E...KF.........I.........S.**

**D.melanogaster**  **RSSRDRERMHKS.EH------.QAS..I.....NTN.S.HKV.EL.NKY.......M.I....Q......**

**D.virilis**  **--------MRQA.DH------.QAS..I.....NTN.T.QKV.EL.NKF.......M.I..H.H......**

**D.pseudoobscura**  **RQSQDRQ-IRGSSER------.KTN..I.....STN.SQEKV.DL.SKY.......M.I..QTH......**

**B.mori**  **SHSPMSSRRRHLGDRVRLLEN.TPS..L.....SLY.TEQQINHI.SKY..VDKV.V.I..KTG......**

**A.mellifera**  **SRSPMSSRRRHVGNR----EN.SPS..L.....SIF.TEQQVHHI.SKY..VERI.V.I..KTGH.K...**

**N.vitripennis**  **SRSPMSSRRRHVGNR----DN.TPS..L.....SIC.SEQSLYHI.SKY..VERVVV.I..KTKRPK...**

220 230 240 250 260 270 280

....|....|....|....|....|....|....|....|....|....|....|....|....|....|

**A.obliqua**  **FIYYQDIADAKAAKDACSGTEIDDRRIRVDYSTTQRPHTPTPGVYMGRYTRRERDH--------------**

**A.fraterculus.sp1** **FIYYQDIADAKAAKDACSGMEIDDRRIRVDYSTTQRPHTPTPGVYMGRYTRRERDH--------------**

**A.fraterculus.sp2** **LIYYQDIADAKAAKDACSGMEIDDRRIRVDYSTTQRPHTPTPGVYMGRYTRRERDH--------------**

**A.fraterculus.sp3** **FIYYQDIADAKAAKDACSGMEIDDRRIRVDYSTTQRPHTPTPGVYMGRYTRRERDH--------------**

**A.fraterculus.sp4** **FIYYQDIADAKAAKDACSGMEIDDRRIRVDYSTTQRPHTPTPGVYMGRYTRRERDN--------------**

**A.grandis**  **FIYYQDIADAKAAKDACSGMEIDDRRIRVDYSTTQRPHTPTPGVYMGRYTRRERDH--------------**

**A.serpentina**  **FIYYEDIADAKAAKDACSGMEIDDRRIRVDYSTTQRPHTPTPGVYMGRYTRRERDH--------------**

**A.sororcula**  **FIYYEDIADAKAAKDACSGMEIDDRRIRVDYSTTQRPHTPTPGVYMGRYTRRERDH--------------**

**A.striata**  **FIYYQDIADAKAAKDACSGMEIDDRRIRVDYSTTQRPHTPTPGVYMGRYTRRERDH--------------**

**A.bistrigata**  **FIYYQDIADAKAAKDACSGMEIDDRRIRVDYSTTQRPHTPTPGVYMGRYTRRERDH--------------**

**A.amita**  **FIYYQDIADAKAAKDACSGMEIDDRRIRVDYSTTQRPHTPTPGVYMGRYTRRERDN--------------**

**B.oleae**  **FIYYEDIADAKAAKDACSGMEIDDRRIRVDYSTTQRPHTPTPGVYMGRQTRREREHN-------------**

**C.capitata**  **FIYYDDIADAKAAKDACSGMEIDDRRIRVDYSTTQRPHTPTPGVYMGRHTRREREYN-------------**

**M.domestica**  **FIYYKHLADAEVARDQCCGQEVDGRRIRVAYSITERPHSPTPGVYRGRSTRSLSQR--------------**

**L.cuprinia**  **FIYYENLADAKAACDNCCGMEIEGRRIRVAYSITERPHTPTPGVYMGRPAKDLRERY-------------**

**D.melanogaster**  **FIYFEKLSDARAAKDSCSGIEVDGRRIRVDFSITQRAHTPTPGVYLGR--QPRGKAPR------------**

**D.virilis**  **FIYFENLGDARVAKDACTGMEVDGRRIRVDYSITQRAHTPTPGVYMGRPSRPLGRRSRER----------**

**D.pseudoobscura**  **FIYFQNVADARVAKDSCCGMEIDNRRIRVDFSITQRPHTPTPGIYMGRSSRNQYGRSGS-----------**

**B.mori**  **FVYFEDMEDAKIAKNECTGMEIDGRRIRVDYSITQRAHTPTPGIYMGKPTISSRGDNGYDRRRDRDDCYY**

**A.mellifera**  **FVYFESLEDAKVAKEQCAGMEIDGRRMRVDYSITQRAHTPTPGIYLGKPTHLHDRG--------------**

**N.vitripennis**  **FVYFESLEDAKVAKEQCSGMAIDGRRIRVDYSITERAHTPTPGIYIGKPTHVCGGGGGSGGGGGGG----**

290 300 310 320 330 340 350

....|....|....|....|....|....|....|....|....|....|....|....|....|....|

**A.obliqua**  **-------DRYRDDYRSR---RRSVTPHNSR-NSYRGDRRRRYDR-----------SRSRSYSPR------**

**A.fraterculus.sp1** **-------..........---..........-.............-----------.........------**

**A.fraterculus.sp2** **-------..........---..........-....A........-----------.........------**

**A.fraterculus.sp3** **-------.....E....---..........-....G........-----------.........------**

**A.fraterculus.sp4** **-------.....D....---..........-....G........-----------.........------**

**A.grandis**  **-------.....D....---......Y.N.-....G........-----------.........------**

**A.serpentina**  **-------.....D....---......H.S.-....G........-----------.........------**

**A.sororcula**  **-------.....D....---......H.S.-....G........-----------.........------**

**A.striata**  **-------.....D....---......Y.S.-....G........-----------.........------**

**A.bistrigata**  **-------.....D....---......Y.S.-....G........-----------.........------**

**A.amita**  **-------.....D....---......H.SR-....G........-----------.........------**

**B.oleae**  **-------.....D..P.---...GSPYHNRS.N..S..S...E.-----------.........------**

**C.capitata**  **-------.....D..P.---...GSPFKNR-.N..N..R...D.-----------.........------**

**M.domestica**  **------------N..Q.---.H.PSPYSRS-------NRD..E.-----------.....H...------**

**L.cuprinia**  **-------RAQKQQLQQQ---QRHYSPVSYSSKSHHSHR-HRYER-----------.....Y...------**

**D.melanogaster**  **---SFSPR---RGRRVYHDR--SASPYDNY-RDRYDYRNDRYDRNLRRSPSRNRYT.N..Y.RS--RSP-**

**D.virilis**  **---DYSTRDTSRSRRRHRDESSSVSPYDSN-RRKYRSR-HRYDR------SR---S.T..Y.RS--RSPR**

**D.pseudoobscura**  **---PCGSRYRDYGSSTTKDSRSRYRDYRNE-RSDREYRNERANRNYPKESSRSRYV.S..V.RSRSRSPV**

**B.mori**  **RGGGGGGGYRERDYYHRGYRHRSPSPHY--------RRTRRYER-----------E.--.Y.PRI-----**

**A.mellifera**  **-----WDGPRRRDSSYRGSYRRSPSPYY-N-----RRRG-RYDR-----------S.SR.Y.P-------**

**N.vitripennis**  **GGSGGWDGQRRRDYNSRGNYRRSPSPYYSN-----RRRSSRYER-----------S.SR.Y.PRF-----**

360

....|....|...

**A.obliqua**  **-----RTRY---***

**A.fraterculus.sp1** **-----....---***

**A.fraterculus.sp2** **-----....---***

**A.fraterculus.sp3** **-----....---***

**A.fraterculus.sp4** **-----....---***

**A.grandis**  **-----....---***

**A.serpentina**  **-----....---***

**A.sororcula**  **-----....---***

**A.striata**  **-----....---***

**A.bistrigata**  **-----....---***

**A.amita**  **-----....---***

**B.oleae**  **-----.A..---***

**C.capitata**  **-----.A..---***

**M.domestica**  **-----.Y..---***

**L.cuprinia**  **-----.Y..RYH***

**D.melanogaster**  **QLRRTSS..---***

**D.virilis**  **KPVRVQS..---***

**D.pseudoobscura**  **RKYRTSS..E--***

**B.mori**  **-----ET.SKG-***

**A.mellifera**  **-------.NI--***

**N.vitripennis**  **-----ES.GIG-***
