# Supplementary material for: The gene transformer-2 of Anastrepha fruit flies (Diptera, Tephritidae) and its evolution in insects
Source: BMC Evol Biol. 2010 May 13;10:140. doi: 10.1186/1471-2148-10-140 (PMC2885393; doi:10.1186/1471-2148-10-140)
Supplement: Additional file 5 — Evolutionary parameters of the Tra2 proteins from insects. Average numbers of amino acid (pAA), total nucleotide (pNT), synonymous (pS) and non-synonymous (pN) nucleotide differences per site, codon bias (Effective Number of Codons, ENC) and Z-test of selection in complete tra-2 genes and independent tra-2 domains from insects. SE, standard error; R, average transition/transversion ratio; n/a, not applicable. a H0: pN = pS; H1: pN <pS. [file 1471-2148-10-140-S5.DOC]

**Table 3: Evolutionary parameters of the Tra2 proteins from insects.**

|  | ***p*_AA_±SE** | ***p*_NT_±SE** | ***p*_S_±SE** | ***p*_N_±SE** | **ENC** | **R** | ***Z*-test^a^** | ***P*-value** |
| --- | --- | --- | --- | --- | --- | --- | --- | --- |
| **Complete** | 0.236±0.020 | 0.359±0.021 | 0.539±0.015 | 0.168±0.014 | 51.643±4.480 | 0.6 | 18.894 | 0.000 |
| **RRM** | 0.196±0.022 | 0.299±0.022 | 0.513±0.018 | 0.129±0.014 | 53.081±6.001 | 0.9 | 17.937 | 0.000 |
| **Linker** | 0.133±0.037 | 0.254±0.049 | 0.514±0.025 | 0.073±0.020 | n/a | 0.7 | 12.120 | 0.000 |
| **RS N-term** | 0.408±0.115 | 0.590±0.095 | 0.448±0.054 | 0.344±0.083 | 51.418±5.732 | 0.4 | 1.220 | 0.113 |
| **RS C-term** | 0.351±0.047 | 0.566±0.061 | 0.456±0.023 | 0.291±0.029 | 39.204±6.777 | 0.8 | 4.040 | 0.000 |
| **Overall RS** | 0.358±0.043 | 0.582±0.054 | 0.482±0.023 | 0.291±0.027 | 46.794±3.742 | 0.4 | 5.407 | 0.000 |
